# Missense Variants in the A Isoform of *FGF13* as a Novel Cause of Paroxysmal Dyskinesia

**DOI:** 10.1002/mds.70256

**Published:** 2026-03-11

**Authors:** Cyril Mignot, Matthildi Athina Papathanasiou Terzi, Claudia Ravelli, Elisabeth Bosch, Xueqin Lin, Adeline Trauffler, Roseline Caumes, Andrew E. Fry, Clementine Fort, Gaelle Gauthe, Regina Trollmann, Thomas Wirth, Mathieu Anheim, Aurélie Méneret, Emmanuel Roze, Jean‐Madeleine de Sainte Agathe, Hailan He, Eleni Panagiotakaki, Gaëtan Lesca, André Reis, Diane Doummar, Thomas Smol, Georgia Vasileiou

**Affiliations:** ^1^ Département de Génétique Médicale, Groupe Hospitalier Pitié‐Salpêtrière et Hôpital Trousseau APHP Sorbonne Université Paris France; ^2^ INSERM, U 1127, CNRS UMR 7225, Sorbonne Université, UPMC Univ Paris 06 UMR S 1127, Institut du Cerveau, ICM Paris France; ^3^ Centre de Référence Déficiences Intellectuelles de Causes Rares, and ERN ITHACA Paris France; ^4^ Department of Pediatric Clinical Epileptology, Sleep Disorders and Functional Neurology, Member of the ERN EpiCare University Hospitals of Lyon (HCL) Lyon France; ^5^ APHP Sorbonne Université Service de Neurologie Pédiatrique, Hôpital Armand Trousseau AP‐HP Paris France; ^6^ Centre de Référence Maladies Génétiques Rares du Système Nerveux Paris France; ^7^ Institute of Human Genetics, Universitätsklinikum Erlangen, Friedrich‐Alexander‐Universität Erlangen‐Nürnberg Erlangen Germany; ^8^ Department of Pediatrics Xiangya Hospital, Central South University Changsha China; ^9^ Pediatric Neurology Department CHU Jeanne de Flandres Lille France; ^10^ CHU Lille Clinique de Génétique, Guy Fontaine Lille France; ^11^ All Wales Medical Genomics Service, Wales Genomic Health Centre Cardiff United Kingdom; ^12^ Division of Cancer and Genetics, School of Medicine Cardiff University Cardiff United Kingdom; ^13^ Paediatrics Neurology Department, Hôpital Femme Mère Enfant, University Hospitals of Lyon (HCL) Lyon France; ^14^ Department of Pediatrics Hospital Center of Metropole Savoie Chambery France; ^15^ Division of Pediatric Neurology, Department of Pediatrics Friedrich‐Alexander‐University of Erlangen‐Nürnberg Erlangen Germany; ^16^ Centre for Rare Diseases Erlangen (ZSEER) Universitätsklinikum Erlangen Erlangen Germany; ^17^ Neurology Department Strasbourg University Hospital Strasbourg France; ^18^ Institute of Genetics and of Molecular and Cellular Biology (IGBMC), INSERM‐U964/CNRS‐UMR7104/Strasbourg University Illkirch‐Graffenstaden France; ^19^ Institute of Neuroscience of Strasbourg University Hospital of Strasbourg Strasbourg France; ^20^ Paris Brain Institute, Sorbonne University, INSERM, CNRS Department of Neurology, DMU Neurosciences, Pitié‐Salpêtrière Hospital, AP‐HP Paris France; ^21^ Laboratoire de Biologie Médicale SeqOIA Paris France; ^22^ Lyon's Neuroscience Research Center Inserm U1028/CNRS UMR 5292 Lyon France; ^23^ Department of Medical Genetics Member of the ERN EpiCARE, University Hospitals of Lyon (HCL), Lyon, France, University Claude Bernard Lyon 1 Lyon France

**Keywords:** caffeine, FGF13A, inactivation particle, methylphenidate, paroxysmal dyskinesia, PxD

## Abstract

**Background:**

Pathogenic variants within the unique N‐terminal inactivation particle of *FGF13* isoform A (*FGF13A*) have so far been associated only with an X‐linked dominant epileptic encephalopathy (DEE).

**Objective:**

The aim was to expand the clinical and molecular spectrum of *FGF13A*‐related disorder.

**Methods:**

We performed exome or genome sequencing of patients with unexplained nonepileptic paroxysmal dyskinesia (PxD).

**Results:**

Four unrelated boys with three novel *de novo* or inherited pathogenic missense variants in *FGF13A* were identified. Variants were also located within the inactivation particle, proximal to the DEE variants. Carrier mothers were unaffected, indicating an X‐linked recessive inheritance. All patients presented with PxD in infancy, consisting of both hyperkinetic and hypokinetic–hypotonic phases, and a variable neurodevelopmental disorder. The frequency of attacks was high (60–100 per day). Treatment with caffeine with or without methylphenidate partially improved PxD in 2 patients.

**Conclusions:**

*FGF13A* variants cause a partially caffeine‐responsive PxD phenotype with variable cognitive impairment, albeit without epilepsy. © 2026 The Author(s). *Movement Disorders* published by Wiley Periodicals LLC on behalf of International Parkinson and Movement Disorder Society.

Paroxysmal dyskinesia (PxD) is a group of movement disorders (MD) characterized by recurrent involuntary episodic movements, including dystonia, chorea, and athetosis. The majority of cases are triggered by a sudden movement change (kinesigenic PxD) or by anxiety, coffee, sleep deprivation, and fatigue (nonkinesigenic PxD), whereas some lack identifiable triggers.^1^ Provoking factors, frequency, and duration are variable. PxD shows a large clinical and genetic heterogeneity. The implementation of genome/exome sequencing enabled the identification of more than 20 known causative genes for isolated PxD.^2^, ^3^ However, the genetic etiology remains unclear in a substantial proportion of PxD cases.^4^

*FGF13*, also known as *FHF2*, is located on the X chromosome and encodes a fibroblast growth homologous factor.^5^ Alternative splicing produces five protein‐coding *FGF13* isoforms, which are expressed in a tissue‐specific manner and have different functions. The major isoform with high expression in the brain is *FGF13A* (NM_004114.5), expressed in both inhibitory and excitatory neurons.^6^

*FGF13A* performs several functions, including the modulation of sodium currents through interaction with different voltage‐gated sodium channels (Na_v_).^7^, ^8^ Within its unique N‐terminus, the *FGF13A* isoform contains an inactivation particle, which mediates rapid, long‐term inactivation of Na_v_ channels (Fig. 1B).^9^

**FIG. 1:**
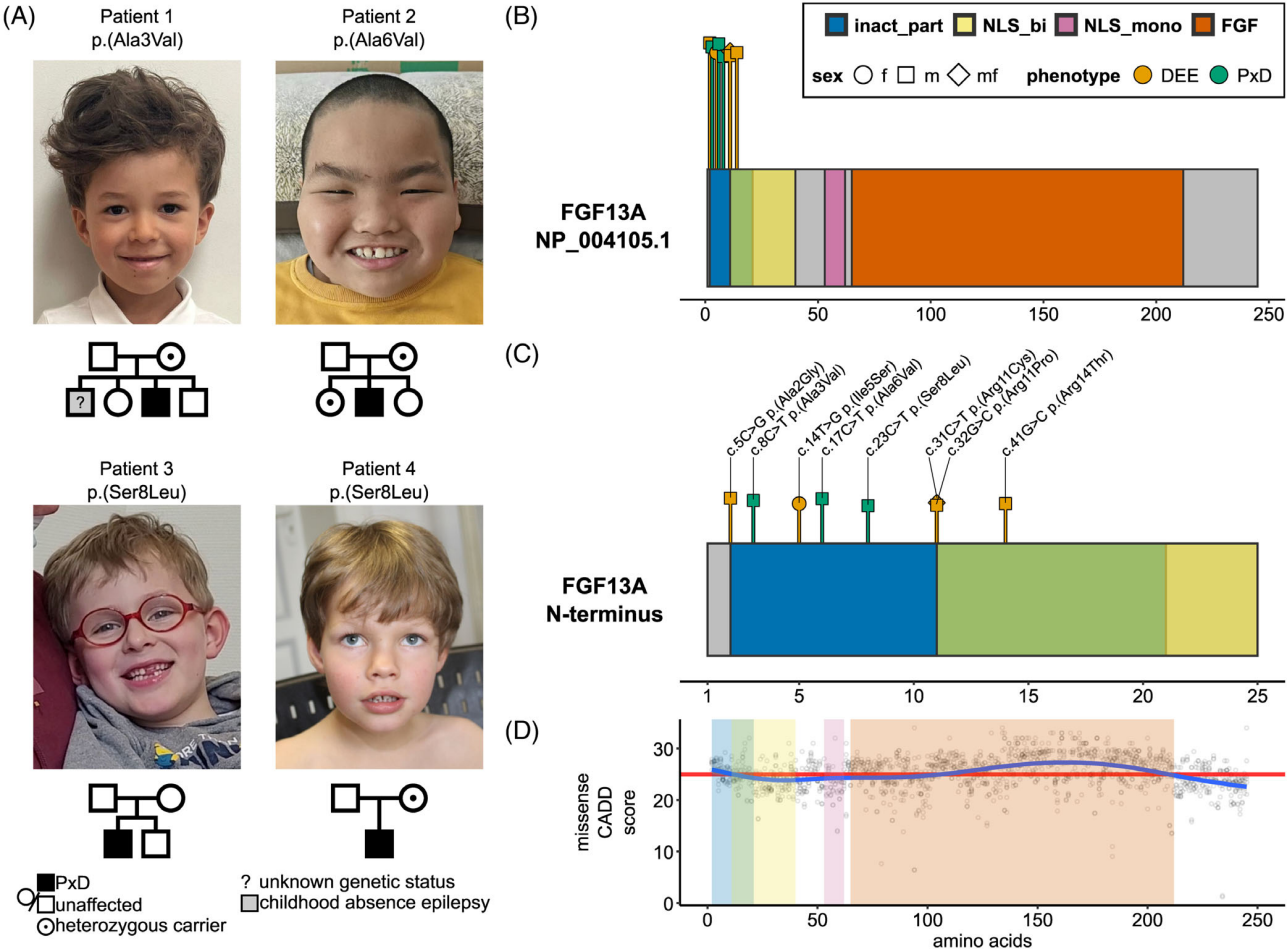
**(A)** Images and pedigrees of the PxD (paroxysmal dyskinesia) patients described here. (**B**) Linear protein structure of FGF13A (NP_004705.1) and its domains: an inactivation particle necessary for sodium channel inactivation (inact_part, amino acids [aa] 2–21, blue), a bipartite, importin‐α‐dependent nuclear localization signal (NLS_bi, aa 11–40, yellow), a monopartite NLS (NLS_mono, aa 53–62, pink), and the fibroblast growth factor core domain (FGF, aa 65–212, dark orange). The inactivation particle and bipartite NLS motifs overlap (aa 11–21, green). Through its FGF domain, FGF13A binds to voltage‐gated sodium channels,^23^ and its N‐ and C‐terminal regions modulate channel activity (see also File S2 sheet domains).^9^, ^24^ (**C**) Enlargement of the N‐terminal inactivation particle (aa 2–21), containing all variants discussed in this report. Lollipops above the protein model show the missense variants causing either developmental and epileptic encephalopathy (DEE, light orange) or PxD (green). The shape of the lollipop heads corresponds to the sex of individuals carrying the variant (circle: only females, square: only males, diamond: both sexes). The lollipop segment length for each variant corresponds to their scaled CADD (Combined Annotation Dependent Depletion) scores.^25^ (**D**) Generalized linear model of scaled CADD scores for all possible missense variants of *FGF13A* (see also File S2 sheet all_missense). The CADD score can indicate variant deleteriousness. It is normalized to all possible single nucleotide variants (SNVs) in the human genome: a value of ≥25 indicates the respective variant is among the top 0.5% of deleterious variants in the genome. The blue line indicates the smoothed CADD values, whereas the red horizontal line represents the recommended cutoff for this score according to ACMG/AMP criteria (framework for interpretation of sequence variants, published by the American College of Medical Genetics and Genomics and the Association for Molecular Pathology).^26^ This analysis indicates that the inactivation particle and a part of the FGF domain are highly conserved and intolerant to missense variants. [Color figure can be viewed at wileyonlinelibrary.com]

Heterozygous or hemizygous pathogenic missense variants in the *FGF13A*‐specific exon 1 have been associated with X‐linked developmental and epileptic encephalopathy 90 (DEE90, MIM* 300070), a disorder characterized by severe developmental delay, absent or limited speech, and drug‐resistant epilepsy. The identified developmental and epileptic encephalopathy (DEE) variants were localized within the inactivation particle.^10^, ^11^, ^12^

Here, we report novel hemizygous missense variants in *FGF13A* identified in 4 unrelated male patients presenting with distinctive PxD, albeit without epilepsy.

## Patients and Methods

All individuals (Fig. 1A) were referred to the neurology departments of pediatric clinics and/or clinical genetic institutes for detailed assessment. Cooperation was established through the web‐based GeneMatcher, ADDIRares, and ERN‐ITHACA platforms. The *FGF13A* variants were identified by trio exome/genome sequencing in research or diagnostic settings in the collaborating centers. Sanger sequencing of patient 2's relatives and X‐inactivation assays were performed using standard methods.^13^ Exome sequencing data of 45 undiagnosed patients with kinesigenic PxD were reanalyzed. More details on the sequencing methods and variant annotation in Figure 1 are presented in File S1, “Supporting Information and Methods.” Written informed consent for publication of clinical and molecular data, photos, and videos was obtained from the patients' legal guardians (Video 1).

**Video 1:** Patient 1: video‐EEG (electroencephalography) recording at 3 years of age. The paroxysmal episode begins with truncal hypotonia lasting 15 seconds. Then generalized hypotonia sets in, accompanied by an opened mouth and hypomimia, without loss of awareness or EEG abnormalities. The episode lasts 3 minutes. Recovery is gradual, over a few tens of seconds: the child can grasp an object, move the limbs, roll onto the side, and then sit up again. Patient 3: clinical examination at 8 years of age. The interictal state is characterized by generalized subcortical myoclonus. The paroxysmal episode begins with a hyperkinetic phase lasting about 20 seconds, marked by generalized hyperkinetic movements involving the face, with an open mouth and associated drooling. Progressively, the hypotonic and hypokinetic phases begin with truncal hypotonia and chewing movements, followed by generalized hypotonia, behavioral arrest, and clenched hands. There is no loss of consciousness. Patient 4: familial video recording at 8 years of age. Interictal state shows mild impairment of fine motor skills. The paroxysmal episode begins abruptly with generalized dyskinetic movements that cause the child to fall to the floor. The child is still able to speak. After ~30 seconds, the hypotonic–hypokinetic phase begins with eyelid drooping, chewing movements, behavioral arrest, and clenched hands. There is no loss of consciousness. Recovery is gradual, lasting a few tens of seconds: the child is able to sit up, although axial hypotonia persists and the hands remain clenched, and he resumes playing (detailed description of the paroxysmal episodes is also provided in File S1).

## Results

### Clinical Description

All 4 patients presented with a nonepileptic paroxysmal MD that began in the first year of life (between 5 and 9 months of age) and persisted throughout childhood up to the last clinical evaluation (Table 1). The severity was similar in patients 2 to 4, whereas patient 1 exhibited a milder form. Brain magnetic resonance imaging and repeated electroencephalography recording between and during the episodes revealed no abnormalities in any of the 4 patients. In patients 2 to 4, the attacks occurred more than 60 times a day and consisted of two phases. The first was a hyperkinetic phase with abrupt onset, presenting with generalized dyskinesia (including the face) and lasting <1 minute, followed by a hypokinetic–hypotonic phase with behavioral arrest and decreased alertness lasting several minutes (Video 1). Both phases occurred in full consciousness. The attacks ended with a gradual return to baseline. In patient 1, the episodes were purely dyskinetic between 6 and 10 months of age, then became less frequent (varying from 2 to 3 per month to 3 per day), and from 1 year of age they were purely hypokinetic–hypotonic. In patients 2 to 4, the attacks were so frequent that no single trigger could be identified: they were both kinesigenic and nonkinesigenic (related to fatigue, psychological stress, fever, or occurred without trigger). In patients 1 to 3, attacks were also observed during sleep.

**TABLE 1:** Clinical and molecular description of the male patients with PxD

Patients ID	1	2	3	4
Sex	M	M	M	M
Ancestry	Caucasian/Algerian	Chinese	Caucasian/African	Caucasian
Current age (yrs)	5.3	10	8	8
*FGF13A* variant (genomic)	chrX(hg38):g.138710996G>A	chrX(hg38):g.138710987G>A	chrX(hg38):g.138710981G>A	chrX(hg38):g.138710981G>A
cDNA change (isoform)	NM_004114.5:c.8C>T	NM_004114.5:c.17C>T	NM_004114.5:c.23C>T	NM_004114.5:c.23C>T
Amino acid change	NP_004105.1:p.(Ala3Val)	NP_004105.1:p.(Ala6Val)	NP_004105.1:p.(Ser8Leu)	NP_004105.1:p.(Ser8Leu)
Inheritance	Maternal	Maternal	*De novo*	Maternal
Genetic testing (panel/WES/WGS)	WGS	WES	WGS	WES
Region	Disordered (inactivation particle)	Disordered (inactivation particle)	Disordered (inactivation particle)	Disordered (inactivation particle)
Other potentially relevant variants	No	*CACNA1F*, maternally inherited, VUS	*IQSEC2*, inherited, VUS	No other relevant variants for this condition; pathogenic compound heterozygous variants in *HFE* gene
Age at diagnosis (yrs)	5	10	8	8
Any relevant family history?	Older brother treated for childhood absence epilepsy; he is now seizure free.	No	No	No
Prenatal findings	Gestational diabetes	No	Gestational diabetes	No
Gestational age (wk)	38 WA + 5 days	40 WA	40 WA	39 WA
Birth parameters weight (g)/length (cm)/OFC (cm)	3250 (41st p.)/48 (15th p.)/36 (82nd p.)	4000 (94th p.)/50 (38th p.)/35 (62nd p.)	3395 (40th p.)/52 (78th p.)/34 (22nd p.)	3360 (39th p.)/51 (35th p.)/31 (<1st p.)
Neonatal period	Uneventful	Uneventful	Uneventful	Uneventful
Developmental delay? yes/no	No	Yes	Yes	Yes
Age at sitting (mo)	6	16	9	NA
Age at walking (mo)	16	Unclear, is currently unable to walk because of extremely frequent dyskinesia	18	42
Age at first words (yrs)	1	6	Delayed	2.5
Current language ability	Full sentences	Full sentences	Full sentences	Simple full sentences with articulation disorder
Intellectual impairment? Formal assessment? Age at assessment and results	No ID	ID is very likely; Gesell (1 yr and 1 mo): adaptive developmental quotient 42 (equivalent age: 4.4 mo), gross motor 27 (2.8 mo), fine motor 44 (4.6 mo), language 53 (5.6 mo), personal‐social 50 (5.2 mo)	No ID, at 5 years old WPPSI VCI 81, FRI 77; 7.5 years old WISC VCI 89, heterogeneous scores, attention, visuo‐constructive and executive difficulties	ID; KABC‐II at 6.5 years old: 5th scale crystallized index, 64; sequential processing scale, 49, simultaneous processing scale, 55; learning ability, 76; planning ability, 67; knowledge, 89
Behavioral disorder?	No	No	No	No
Age at onset of the current episodes (mo)	6	8	5	8–9
Description of paroxysmal phenomena	6 mo: paroxysmal tremor of head and upper limbs with nystagmus, duration 2 min at max; from the age of 12 mo: hypotonia of four limbs with hypotonia and hypokinesia, behavioral arrest, chewing, drooling, no loss of awareness, duration 5 min at the maximum	Two phases: several instances of eye blinking, lip smacking, twisting movements of the upper limbs involving internal and external rotations, or chorea‐like manifestations, followed by hypotonia and hypokinesia, without loss of consciousness	Two phases: generalized dyskinesia involving the face (20–25 seconds), followed by hypotonia and hypokinesia, behavioral arrest, clenched hands (1–2 minutes), no loss of awareness	Two phases: dyskinesia of upper limbs and face followed by hypotonia and hypokinesia, behavioral arrest, clenched hands and chewing, no loss of awareness; frequent prodromal phase with eye‐rolling, behavioral changes
Facial dyskinesia?	No, facial akinesia with drooling	Yes	Yes	Yes
Triggers of episodes?	Nonkinesigenic (tiredness, illnesses, fever)	Kinesigenic and nonkinesigenic	Kinesigenic and nonkinesigenic (emotional stress, tiredness, fever, heat, or no trigger)	Kinesigenic and nonkinesigenic (stress conditions)
Nocturnal spells?	Yes	Yes	Yes	No
Duration of episodes?	5 minutes	5–10 minutes	2–5 minutes; one longer episode after awakening (20 minutes)	2–5 minutes, longer episodes after awakening (10–20 minutes)
Frequency of episodes?	First twice a day, then fluctuation, then 2 to 3 per month but 20 per day with fever; currently 2 to 3 per day	Approximately 60–70 per day (6 times per hour)	>100 per day before treatment	Approximately 60 per day
Treatments and response	Flunarizine 2 mg/day for 1 mo ineffective	Flunarizine, oxcarbazepine oral solution 12.75 mg/kg/day and levodopa/benserazide ineffective; caffeine base currently 5.2 mg/kg/day shortened the intensity and reduced the frequency of hyperkinetic phase episodes	Levodopa/benserazide ineffective; carbamazepine 10 mg/kg/day reduced the number of episodes but increased its severity; caffeine base currently 4.3 mg/kg/day shortened the hypotonic phase and reduced slightly the frequency of episodes; association with immediate‐release methylphenidate 10 mg/day allowed the disappearance of the hypokinetic phase of certain attacks, without clear effect on the hyperkinetic phase. Switching to the modified‐release methylphenidate (10 mg/day) reduced the PxD episodes by 50%	Levetiracetam, topiramate, valproate, carbamazepine, ketogenic diet not effective; deep brain stimulation: no improvement. clonazepam slightly reduced the episodes but they lasted longer; currently no medication is administered
Epilepsy ruled out?	Yes	Yes	Yes	Yes
Brain MRI	Normal	Normal	Normal	Normal
Baseline neurological examination (between the episodes)	Normal	Generalized dystonia and hyperreflexia and positive ankle clonus	Generalized hypotonia with generalized action dystonia and myoclonus, facial dyskinesia	Uncoordinated and wide‐based walking, fine motor skills mildly restricted
Height (cm/SD); weight (kg/SD); OFC (cm/SD) at last examination	106/0; 16.6/0; 52.5/+1	142/−0.5; 57.5/+1; 57/+3	136/+2; 24/+1; 51.5/−0.5	132/+0.24; 25/−0.6; 51.5/−1.1

Abbreviations: cDNA, complementary DNA; FRI, fluid reasoning index; ID, intellectual disability; KABC‐II, kaufman assessment battery for children, second edition; MRI, magnetic resonance imaging; NA, not available; OFC, occipitofrontal circumference; PxD, paroxysmal dyskinesia; SD, standard deviation; WES, whole exome sequencing; WGS, whole genome sequencing; WPPSI, wechsler preschool and primary scale of intelligence; VCI, verbal comprehension index; VUS, variant of unknown significance; WA, weeks of amenorrhea.

Several pharmacologic approaches were ineffective, including ketogenic diet and deep brain stimulation in patient 4. Only caffeine exhibited partial efficacy in 2 patients, reducing the intensity and frequency of the hyperkinetic phase in one and the duration of the hypotonic phase in the other. In the latter, combination with methylphenidate led to a significant reduction in attack frequency. This treatment has not yet been administered to the other 2 patients (see also Table 1 and File S1).

Baseline examination between episodes of patients 2 to 4 revealed motor impairment, including dystonia in 2 of them and uncoordinated walking in patient 4. Patient 1 had age‐appropriate neurological findings. Patients 2 to 4 exhibited developmental delay, including learning difficulties or mild intellectual impairment (Table 1; File S1).

### Genetic Analysis

We detected three amino acid (aa) substitutions located in exon 1 of the *FGF13A* isoform, which is absent from the other *FGF13* transcripts. These had not previously been described in the literature or listed in international patients' databases, were absent from the public database gnomAD, and were predicted as pathogenic by prediction algorithms (File S2 sheet cohort_variants). Patients 1 and 2 carried the maternally inherited variants p.(Ala3Val) and p.(Ala6Val), respectively; the heterozygous mothers were healthy. The sister of patient 2 was also an unaffected carrier. The same variant p.(Ser8Leu) was identified in patients 3 and 4, occurring *de novo* in the former and being inherited from the unaffected mother in the latter (Fig. 1A–C). Analysis of the healthy carrier mothers of patients 1 and 4 revealed a random X‐inactivation status in peripheral lymphocytes with a ratio of 68% and 67%, respectively.

Reanalysis of the exome data of 45 patients with genetically undiagnosed kinesigenic PxD did not detect any additional *FGF13A* variants.

## Discussion

To date, pathogenic variants in *FGF13A* have been reported only in patients with severe DEE.^10^ This study is the first to describe a novel *FGF13A*‐associated nonepileptic PxD with peculiar characteristics and variable cognitive impairment.

*FGF13A*‐related PxD begins in the first year of life, is mainly biphasic, lasts several minutes (3–20 minutes), and exhibits a very high frequency (several dozen per day). Triggers are diverse, kinesigenic, and nonkinesigenic, and episodes may occur during sleep. The early onset and high frequency of PxD, facial involvement, and sleep onset are reminiscent of individuals with gain‐of‐function variants in *ADCY5* ^14^ and biallelic loss‐of‐function variants in *PDE2A*.^15^ The sequence of a hyperkinetic phase followed by a hypokinetic–hypotonic phase in 3 of 4 patients has previously been reported for *PDE2A* variants.^15^ The isolated hypokinetic–hypotonic phase, observed in 1 patient, resembles the paroxysmal phenomena associated with *KCNMA1* variants or the plegic attacks linked to *ATP1A3* or *RHOBTB2* variants.^16^, ^17^ Another differential diagnosis is narcolepsy of autoimmune origin, which may begin in children with episodes of cataplexy associating “negative” (hypotonia) and “active” (ranging from perioral movements to dyskinetic‐dystonic movements) motor disturbances.^18^ However, in narcolepsy, these phenomena occur simultaneously, are triggered by laughter and emotions, and typically manifest later, from the age of 6 years.

*FGF13A*‐associated PxD did not respond to conventional treatments. Due to the efficacy of caffeine and methylphenidate in *ADCY5*‐related dyskinesia,^19^, ^20^ we suggested this combination to our patients. Caffeine exhibited partial effectiveness in 2 patients. Notably, the combination with methylphenidate enhanced the effect of caffeine treatment (File S1). Our findings add to the ongoing evidence for the therapeutic effect of caffeine in patients with genetic PxD. However, a more systematic evaluation of these treatments is warranted.

By exome and genome sequencing, we identified 3 novel missense variants in exon 1 of *FGF13A* in 4 patients with PxD. These variants differ from those previously associated with DEE but cluster similarly to the latter within the first 21 residues at the N‐terminus, which encode the inactivation particle (Fig. 1B,C). Interestingly, this domain along with a part of the FGF domain is the most highly conserved in *FGF13A*, and is intolerant to aa substitutions (Fig. 1D).

Despite the clustering, the phenotypic divergence indicates functional differences between DEE and PxD variants. To date, 7 male and 3 female patients with DEE from 6 unrelated families, harboring 5 distinct *FGF13A* pathogenic missense variants, have been described (Fig. 1B).^10^, ^11^, ^12^ None of these variants were inherited. *De novo* occurrence, low‐grade somatic mosaicism, or gonadal mosaicism in the unaffected mother were observed. Both males and females were affected, suggesting an X‐linked dominant inheritance. In contrast, all affected individuals with the PxD variants described here are males. Three of four aa substitutions were inherited from heterozygous unaffected mothers, indicating X‐linked recessive inheritance.

We considered nonrandom X‐chromosome inactivation (XCI) as a possible explanation for why female carriers of PxD variants are unaffected. An XCI assay in peripheral lymphocytes of 2 healthy carrier mothers did not identify skewing toward the normal allele. However, we cannot rule out the possibility that blood is not representative of all tissues.

Fry et al. showed that the DEE variants p.(Arg11Cys) and p.(Arg14Cys) (Fig. 1B,C) reduce or completely disrupt the ability of the inactivation particle to mediate long‐term inactivation of Na_v_1.6 in excitatory neurons, resulting in increased excitability and seizures.^10^ A loss‐of‐inhibitory function was suggested. A recent study in *Fgf13*‐knockout mice confirmed the aberrant long‐term inactivation of Na_v_1.6 channels but suggested that this is not the primary mechanism triggering seizures. Ablation of *Fgf13* in inhibitory interneurons reduced K^+^ channel currents, leading to a depolarization block and reduced inhibitory drive, thus causing seizures.^21^ Although further experimental studies are needed to understand the effects of DEE variants, aberrant regulation of Na^+^/K^+^ voltage‐gated channels in neurons appears to be one of the driving mechanisms of DEE.

The three PxD‐associated *FGF13A* variants have not yet been functionally investigated. Because they are also located in the inactivation particle, dysregulation of Na_v_ channel activity could underly both the PxD and DEE clinical entities. FGF13A has aliphatic (residues 1–10) and cationic (residues 11–20) regions involved in modulating and maintaining the long‐term inactivated state of sodium channels.^22^ DEE variants remove key cationic or aliphatic residues, which reduces the ability of FGF13A to bind sodium channels and cause long‐term block. In contrast, all PxD substitutions have larger aliphatic side chains. The increased hydrophobicity of these residues may enhance the interaction between FGF13A and sodium channels, leading to reduced neurotransmission and hypokinetic symptoms. In addition, the milder clinical presentation of the PxD patients may be attributed to a milder impairment of FGF13A protein function compared to the DEE variants. Nevertheless, it cannot be excluded that *FGF13* isoform A exerts additional roles and functions in neuronal cells that are differentially impacted by the PxD and DEE pathogenic variants.

## Author Roles

Conception: C.M., C.R., A.E.F., D.D., G.V.; project organization: C.M., G.V.; molecular and exome‐genome analysis: T.W., A.M., J.‐M.S.A., G.L., H.H., T.S., G.V.; clinical evaluation and management of patients: C.M., M.A.P.T., C.R., X.L., A.T., R.C., C.F., G.G., R.T., T.W., M.A., A.M., E.R., E.P., A.R., D.D., G.V.; writing of clinical reports: C.M., M.A.P.T., C.R., X.L., D.D., G.V.; annotation and variant analysis: E.B.; figure creation: E.B., G.V.; writing—original draft: C.M., C.R., E.B., D.D., M.A.P.T., G.V.; review and editing: A.E.F., A.R.; all coauthors read, corrected, and approved the manuscript.

## Financial Disclosures and Conflicts of Interest

Author disclosures are available in the Supporting Information.

**Relevant conflicts of interest/financial disclosures:** C.M.: none. M.A.P.T.: none. C.R.: none. E.B.: none. X.L.: none. A.T.: none. R.C.: none. A.E.F.: none. C.F.: none. G.G.: none. R.T.: none. T.W.: none/was funded by the French Society of Neurology. M.A.: none. A.M. none. E.R.: none/was funded by a research grant from the Foundation Maladies Rares. J.‐M.S.A.: none. H.H.: none. E.P.: none. G.L.: none. A.R.: none. D.D.: none. T.S.: none. G.V.: none.

## Full financial disclosures of all authors for the previous 12 months

C.M.: none. M.A.P.T.: none. C.R.: none. E.B.: none. X.L.: The Fundamental Research Funds for the Central Universities of Central South University (2024ZZTS0923 to LXQ). A.T.: none. R.C.: none. A.E.F.: none. C.F.: none. G.G.: none. R.T.: none. T.W.: none. M.A.: none. A.M. received honoraria for speeches from Merz‐Pharma and AbbVie, and travel funding from Elivie and Merz‐Pharma. E.R. received honorarium for speech from Orkyn, Aguettant, Elivie, Merz‐Pharma, Janssen, Teva, and Everpharma and for participating in advisory boards for Merz‐Pharma, Elivie, Teva, and Bial. He also received research support from Merz‐Pharma, Orkyn, Elivie, Everpharma, and Aguettant. J.‐M.S.A.: none. H.H.: none. E.P.: received honoraria for speeches from Eisai, Jazz Pharmaceuticals, and UCB. G.L.: none. A.R.: none. D.D.: none. T.S.: none. G.V.: none.

## Supporting information


**File S1.** Supplementary data with Supporting Information and Methods, detailed clinical semiology, and treatment as well as references.

**File S2.** Excel file containing information on FGF13A protein domains (“domains”), genetic information, and prediction scores for the variants included in this study (“cohort_variants”), and for all possible missense variants in *FGF13A* (“all_missense”) used for the creation of Figure 1D. A detailed description of the file content is included in the datasheet “readme”.

## Data Availability

The data that support the findings of this study are available in the Supporting Information of this article.
