# Supplementary material for: Missense Variants in the A Isoform of FGF13 as a Novel Cause of Paroxysmal Dyskinesia
Source: Mov Disord. 2026 Mar 11;41(7):1884–92. doi: 10.1002/mds.70256 (PMC13388009; doi:10.1002/mds.70256)
Supplement: Supplementary file 2 — File S1. Supplementary data with Supporting Information and Methods, detailed clinical semiology, and treatment as well as references. [file MDS-41-1884-s002.docx]

**SUPPLEMENTARY DATA**

**Supplementary Material and Methods**

**Patients**

All patients were repeatedly evaluated by pediatric neurologists with expertise in movement disorders and/or clinical geneticists. Medical records and videos were evaluated by pediatric neurologists with expertise in MD. Male Patient 1 was referred to the Department of Pediatric Clinical Epileptology at University Hospital in Lyon*,* male Patient 2 to the Department of Pediatrics at Xiangya Hospital in Changsha, male Patient 3 to the neuropediatric Department at Armand Trousseau Hospital in Paris, and male Patient 4 to the Department of Paediatric Neurology and the Human Genetics Institute at University Hospital Erlangen in Germany for detailed clinical and developmental assessment.

**Genome and Exome sequencing**

Trio genome sequencing of Patient 1 and his parents was performed at the AURAGEN laboratory (LBMMS Auragen, Auvergne Rhône-Alpes) on a NovaSeq6000 sequencer (Illumina) following the recommendations of the PFMG. Sequencing reads were aligned to the GRCh38p13 full assembly using bwa 0.7+. Over 90% of the targeted genomic regions were sequenced at a depth of more than 30x. Variants were called by several algorithms, including GATK4+, Bcftools1.10+, Manta1.6+, CNVnator0.4+, and annotated using the variant effect predictor. Detected variants were prioritized using in-house procedures.

Trio exome sequencing of Patient 2 and his parents was performed using the DNBSEQ-T7 sequencer (MGI) and the xGen® Exome Research Panel v2.0 (IDT) to target the coding regions and selected non-coding regions of 19.396 human genes, covering a 51 Mb interval. Alignment of sequencing reads to the GRCh37/hg19 reference genome was performed using BWA Sequencing. Over 99% of the targeted genomic regions were sequenced at a depth of >20x. Rare variants were assessed using an in-house analysis tool developed by Beijing Chigene Translational Medicine Research Center Co., Ltd. SNP and Indel calling and filtering was performed using GATK to obtain high-quality variants. Exome-based CNV analysis was performed using the ExomeDepth software (GATK).

Trio genome sequencing of Patient 3 and his parents was performed at the SeqOIA laboratory (LBMS SeqOIA, Paris, France) on a NovaSeq6000 instrument (Illumina) following the recommendations of the PFMG as previously described (1).

Trio exome sequencing of Individual 4 and his parents was performed on an Illumina HiSeq 2500 sequencer using the Twist Human Core Exome Enrichment Technology (Twist Bioscience Inc) to cover a target of greater than 99% of all protein-coding genes at a depth of >20x. Sequencing reads were aligned to the GRCh37/hg19 reference genome with BWA-MEM and variants were called by several algorithms, including GATKHap, GATKUG, CASAVA, SNVer, freeBayes, and Platypus. Data were assessed for rare variants using an in-house analysis tool (2). In total, 1,557 genes associated with neurodevelopmental delay (SysNDD database, May 2022 version (3)), 280 genes linked to choreoathetosis and chorea (HP:0001266 and HP:0002072), and 287 genes associated with dyskinesia (HP:0100660; status as of February 8, 2022) were evaluated. Exome-based copy number variant (CNV) analysis was performed using the ExomeDepth Software (GATK).

**Variant annotation**

All variants were standardized according to HGVS recommendations (hg38; NM_004114.5; NP_004105.1) (4) and annotated using the variant effect predictor (5). To assess protein conservation, all possible missense variants for *FGF13A* were calculated and scored using Combined Annotation Dependent Depletion (CADD) v1.7 (6), and their scaled CADD scores were plotted along the linear protein representation. NLS sequences were identified with NLSmapper (7), which searches for classical (monopartite) and bipartite importin-α-dependent NLSs. Plots were generated with R version 4.4.3 (8) using RStudio version 2024.12.1 (9).

**X chromosome inactivation (XCI) analysis**

X-inactivation status was determined using the well-established HUMARA assay (10). This assay is a methylation-sensitive fragment analysis of a polymorphic CAG repeat in exon 1 of the androgen receptor (*AR*) gene. The X-chromosome inactivation ratio was calculated as previously reported (11). An XCI ratio of 75% or more was regarded as a skewed XCI. More specifically, X-inactivation ratios between 75:25 and 90:10 are considered moderately skewed, whereas ratios ≥90:10 are reported as highly skewed (11).

**Detailed clinical descriptions**

**Patient 1** was a 5-year-old male born to healthy parents with normal birth parameters after a pregnancy with gestational diabetes mellitus.

Family history disclosed a similar phenotype up to the age of 10 in the maternal grandfather and two of his brothers. However, this information is based on the observation of a family relative. Clinical records or further clinical data on these individuals were not available, as they live in another country or have no contact with our patient’s family. The patient’s older brother presented with childhood absence epilepsy and is currently seizure free without any medication. His younger brother had simple febrile seizures. Paroxysmal episodes started when Patient 1 was 6 months old and were characterized by truncal hypotonia, tremor of the head and upper limbs, sometimes associated with nystagmus. Episodes lasted up to two minutes. They occurred every day with a maximum of 10 episodes per day until the age of 10 months. Rarely episodes occured during sleep. Repeated video EEGs and brain MRI were unremarkable. From the age of 1 year old, the spells occurred daily and changed into paroxysmal attacks of generalized hypotonia and hypokinesia, opened mouth, hypomimia, chewing movements, and drooling. Episodes lasted for about five minutes without loss of awareness. Tiredness or respiratory illness triggered these paroxysms. The patient experienced intervals of ten days free of episodes, but frequency increased during fever up to 20/day. At 5 years old, Patient 1 had normal neurodevelopment, no motor issues or learning difficulties in school were reported. Baseline clinical examination is normal, without dysmorphic features or neurological signs. Flunarizine was not effective.

Trio genome sequencing revealed the maternally inherited hemizygous variant NM_004114.5: c.17C>T p.(Ala3Val) in *FGF13A.*

**Patient 2** was a 10-year-old male, the third child in the family. He was born at 40 weeks of gestation to non-consanguineous healthy parents after an uncomplicated pregnancy and delivery. Family history was unremarkable. At 8 months of age, the patient began experiencing episodic dyskinesia without any apparent triggers. The spells started with several instances of eye blinking, lip smacking, twisting movements of the upper limbs involving internal and external rotation, or chorea-like manifestations, followed by generalized weakness with drooling. During these spells, consciousness was preserved, with maintained eye contact and emotional responsiveness to verbal stimuli. The spells occurred 60-70 times per day, lasting 5-10 minutes each. Some episodes occured during sleep, though not frequently (approximately 1-2 times).

Patient 2’s developmental milestones were delayed: he achieved head control at 6 months, rolling over at 12 months, independent sitting at 18 months, and supported standing at 2 years and 8 months. He spoke his first words at the age of 6 years old. He has never been able to walk independently. At 13 months, the Gesell Developmental Schedules indicated an adaptive developmental quotient of 42 (equivalent age 4.4 months), gross motor function of 27 (equivalent age 2.8 months), fine motor function of 44 (equivalent age 4.6 months), language function of 53 (equivalent age 5.6 months), and personal-social function of 50 (equivalent age 5.2 months). He has never attended school and is unable to read or count and has not undergone any further intelligence testing. His parents described him as having a good memory, remembering all the cartoons and people he had seen.

Neurological examination revealed generalized dystonia, hyperreflexia, and a positive ankle clonus. Brain MRI wasnormal, as were EEG, auditory brainstem response, and electromyography (EMG). Cerebrospinal fluid biochemical analysis and metabolic screening of blood and urine were also unremarkable. Treatment with levodopa-benserazide, oxcarbazepine (12.75 mg/kg/day), and flunarizine did not significantly improve PxD. After comprehensive cardiac evaluation, caffeine was initiated at 50 mg twice daily, with a weekly dose increase of 50 mg. Upon reaching 100 mg three times daily (5.2 mg/kg/day), parents reported a reduction in the intensity and frequency of hyperkinetic phase episodes. No significant adverse effects have been observed to date.

Trio exome sequencing identified the maternally inherited hemizygous variant NM_004114.5: c.17C>T p.(Ala6Val) in *FGF13A*. Segregation analysis of family members via Sanger sequencing revealed that the maternal grandmother, aunt, and younger sister did not have the *FGF13* variant, while the unaffected older sister was carrier.

**Patient 3** was an 8-year-old male born to healthy parents with normal growth parameters, perinatal period and family history. He had feeding difficulties from the age of 3 months, and at the age of 5 months, paroxysmal events appeared with a phenomenology that remained unchanged over time. PxDs typically last between 2 and 5 minutes; however, a longer episode tends to occur shortly after awakening, with a duration of about 20 minutes. The spells start with a hyperkinetic phase lasting 20-25 seconds, characterized by generalized hyperkinetic movements that include the face, with open mouth, associated to drooling. Progressively, a hypotonic and hypokinetic phase begins and lasts 1 to 5 minutes, with behavioral arrest, clenched hands, eyelid drooping, but no loss of awareness. The child gradually returns to baseline status in less than 1 minute.

Patient 3 remains fully conscious during the complete episode and is able to say simple words during the hyperkinetic phase and at the beginning of the hypotonic-hypokinetic stage. Biphasic PxD is both kinesigenic and nonkinesigenic (fatigue, psychological stress, fever or no trigger). More than 100 episodes occur every day. Some spells occur during sleep. Repeated EEGs and brain MRI were normal.

Patient 3 had a mild neurodevelopmental delay with first steps achieved at 18 months and delayed speech. At the age of 8 years, Patient 3 has mainly a motor impairment and attends a special school for children with motor disability. He uses a wheelchair for long-distance walks and his fine motor skills are severely impaired. His reading ability is that of a beginner. Psychometric assessment performed at 7.5 years old using the WISC-V disclosed a normal-low VCI (89) excluding intellectual disability, while the VSI, FRI, WMI, and PSI could not be assessed because of intra-indices discrepancies. Clinical examination between the episodes revealed generalized hypotonia associated with generalized action dystonia myoclonus, and facial dyskinesia.

Treatment with L-Dopa, acetazolamide and flunarizine were ineffective. Carbamazepine 10 mg/kg/day reduced the number of episodes but increased their severity. Methylphenidate 10 mg/day was introduced firstly at the age of 3 years: it reduced the duration of the hypotonic-hypokinetic phase but was ineffective on dyskinesia and induced mood disorder, so it was discontinued. Caffeine was introduced at the age of 7 years, at the dose of 4.3 mg/kg/day; it reduced the duration of the hypotonic phase and slightly the frequency of the PxD. Increasing the caffeine dose to 8 mg/kg/day did not result in any additional clinical benefit. At the age of 8 years, combination with immediate-release methylphenidate 10 mg/day allowed the disappearance of the hypokinetic phase of certain attacks, without clear effect on the hyperkinetic phase. Perioral dyskinesia appeared one hour after treatment administration. After three months, switching to the modified-release methylphenidate was better tolerated and led to a significant improvement, with a 50% reduction in the frequency of PxD attacks.

Trio genome sequencing identified the *de novo* hemizygous variant NM_004114.5: c.23C>T p.(Ser8Leu) in *FGF13A*.

**Patient 4** was an 8-year-old boy born at 39 weeks of gestation to non-consanguineous, healthy parents. He is the only child in the family. The family history was unremarkable. Pregnancy, delivery and neonatal period were uneventful. Birth weight was 3360 g (-0.29 SD), length 51 cm (-0.39 SD) and occipitofrontal circumference (OFC) 31 cm (-3.31 SD).

At the age of 3-4 months the parents noticed a head and foot twisting during baby swimming lessons. At 8-9 months of age the first paroxysmal episodes were reported. Suspicion of epilepsy was raised, however, routine, prolonged, and sleep EEGs showed repeatedly negative results. Brain MRI showed no abnormalities. At the age of 1.5 years PxD was diagnosed. During the prodromal phase eye-rolling, behavioral changes including aggressiveness, throwing of objects, biting, verbal insults, and overall an irrational behavior are observed. The spells begin with generalized dyskinetic movements, including the face, followed by a hypotonic-hypokinetic phase with behavioral arrest, chewing movements, and clenched hands. During the hyperkinetic phase, Patient 4 can still speak or play. As he enters the hypotonic phase he speaks less and more slowly. As this phase progresses, he is unable to communicate or respond for about 20 to 30 seconds, but maintains eye contact and does not lose consciousness. Patient 4 has approximately 60 attacks per day, each lasting 2-5 minutes. However, the first episode of the day, shortly after waking up, can last up to 10-20 min. The attacks are kinesigenic or occur without identifiable triggers; the parents observed that stress could increase the frequency and duration of PxD.

Treatment with various antiepileptic drugs including levetiracetam, topiramate, and valproate, was not effective. Furthermore, Patient 4 was treated with carbamazepine, but the therapy was discontinued due to complications. A ketogenic diet as well as a deep brain stimulation (DBS) did not bring any improvement. The last administered medication was clonazepam, which slightly reduced the frequency of the PxD episodes, but they lasted longer. Currently he is not under treatment.

Patient 4 displayed developmental delay. He sat independently with delay and he was able to walk at 3.5 years of age. Mild truncal hypotonia was observed in early childhood. At the age of 8, gross motor skills were mildly impaired with an unsteady and wide-based walking between the PxD episodes. Nevertheless, he was still able to run, climb and ride a bicycle. He used foot orthoses due to bilateral pes planus und pes valgus. Mild fine motor deficits were also reported. Patient 4 spoke first words at 2.5 years of age. By 5.8 years, he was using three-word sentences and by 8 years of age, he was able to speak simple full sentences, albeit with articulation difficulties. The receptive language was better. At the time of the last clinical assessment Patient 4 was unable to read. Cognitive assessment using the KABC-II with 6.5 years showed an intelligence quotient (IQ) of 64 (5th scale crystallized index 64; sequential processing scale 49, simultaneous processing scale 55, learning ability 76, planning ability 67, knowledge 89), suggesting mild intellectual disability. He is now attending a special school.

Trio exome sequencing revealed the maternally inherited hemizygous variant NM_004114.5: c.23C>T p.(Ser8Leu) in *FGF13A.*

**Supplementary references**

1. Tusseau M, Eyries M, Chatron N, Coulet F, Guichet A, Colin E, et al. Genome sequencing identify chromosome 9 inversions disrupting ENG in 2 unrelated HHT families. European Journal of Medical Genetics. 2024 Apr;68:104919.

2. Hauer NN, Popp B, Schoeller E, Schuhmann S, Heath KE, Hisado-Oliva A, et al. Clinical relevance of systematic phenotyping and exome sequencing in patients with short stature. Genetics in Medicine. 2018 Jun;20(6):630–8.

3. Kochinke K, Zweier C, Nijhof B, Fenckova M, Cizek P, Honti F, et al. Systematic Phenomics Analysis Deconvolutes Genes Mutated in Intellectual Disability into Biologically Coherent Modules. The American Journal of Human Genetics. 2016 Jan;98(1):149–64.

4. Hart RK, Fokkema IFAC, DiStefano M, Hastings R, Laros JFJ, Taylor R, et al. HGVS Nomenclature 2024: improvements to community engagement, usability, and computability. Genome Med. 2024 Dec 20;16(1):149.

5. McLaren W, Gil L, Hunt SE, Riat HS, Ritchie GRS, Thormann A, et al. The Ensembl Variant Effect Predictor. Genome Biol. 2016 Dec;17(1):122.

6. Schubach M, Maass T, Nazaretyan L, Röner S, Kircher M. CADD v1.7: using protein language models, regulatory CNNs and other nucleotide-level scores to improve genome-wide variant predictions. Nucleic Acids Research. 2024 Jan 5;52(D1):D1143–54.

7. Kosugi S, Hasebe M, Matsumura N, Takashima H, Miyamoto-Sato E, Tomita M, et al. Six Classes of Nuclear Localization Signals Specific to Different Binding Grooves of Importin α. Journal of Biological Chemistry. 2009 Jan;284(1):478–85.

8. R Core Team. R: A Language and Environment for Statistical Computing [Internet]. Vienna, Austria: R Foundation for Statistical Computing; 2025. Available from: https://www.R-project.org/

9. Posit Team. RStudio: Integrated Development Environment for R [Internet]. Boston, MA: Posit Software, PBC; 2025. Available from: http://www.posit.co/

10. Allen RC, Zoghbi HY, Moseley AB, Rosenblatt HM, Belmont JW. Methylation of HpaII and HhaI sites near the polymorphic CAG repeat in the human androgen-receptor gene correlates with X chromosome inactivation. Am J Hum Genet. 1992 Dec;51(6):1229–39.

11. Lau AW, Brown CJ, Peñaherrera M, Langlois S, Kalousek DK, Robinson WP. Skewed X-Chromosome Inactivation Is Common in Fetuses or Newborns Associated with Confined Placental Mosaicism. The American Journal of Human Genetics. 1997 Dec;61(6):1353–61.
